# Co-culture engineering: a promising strategy for production of engineered extracellular vesicle for osteoarthritis treatment

**DOI:** 10.1186/s12964-023-01467-9

**Published:** 2024-01-10

**Authors:** Abazar Esmaeili, Samaneh Hosseini, Mohamadreza Baghaban Eslaminejad

**Affiliations:** 1 Department of Stem Cells and Developmental Biology, Cell Science Research Center, Royan Institute for Stem Cell Biology and Technology, ACECR, Tehran, Iran; 2 Department of Cell Engineering, Cell Science Research Center, Royan Institute for Stem Cell Biology and Technology, ACECR, Tehran, Iran

**Keywords:** Co-culture engineering, Extracellular vesicle engineering, EV therapy, Mesenchymal Stem Cell, Chondrocyte, Osteoarthritis

## Abstract

The therapeutic effects of extracellular vesicles (EVs) have been identified as a significant factor in intercellular communication in different disease treatments, including osteoarthritis (OA). Compared to the conventional approaches in treating OA, EV therapy is a non-invasive and cell-free method. However, improving the yield of EVs and their therapeutic effects are the main challenges for clinical applications. In this regard, researchers are using the EV engineering potential to overcome these challenges. New findings suggest that the co-culture strategy as an indirect EV engineering method efficiently increases EV production and quality. The co-culture of mesenchymal stem cells (MSCs) and chondrocytes has improved their chondrogenesis, anti-inflammatory effects, and regenerative properties which are mediated by EVs. Hence, co-culture engineering by considerable systems could be useful in producing engineered EVs for different therapeutic applications. Here, we review various co-culture approaches, including diverse direct and indirect, 2D and 3D cell cultures, as well as static and dynamic systems. Meanwhile, we suggest and discuss the advantages of combined strategies to achieve engineered EVs for OA treatment.

## Back﻿ground

Extracellular vesicle (EV) therapy is a promising nascent cell-free therapeutic approach [1–3]. Despite the rapid advances in the EV research, their clinical applications still remain challenges [4]. The advent of EV engineering either directly or indirectly will be able to remove pitfalls such as low rates of EV production and cargo enrichment [5]. The co-culture engineering approach is a type of indirect EV engineering that may lead to the high-yield EV production [5]. Co-culture engineering recapitulates the in vivo phenomena, in order to find their cell interactions and mechanisms [6]. In addition, simultaneous culture of different cell types could lead to the secretion of a mixture of engineered EVs [6]. The co-culture engineering would be a practical suggestion for the design and engineering of artificial cell niches to imitate their natural conditions (such as what has been started for a stem cell niche design) in order to harvest co-cultured EVs (Co-EVs) [7, 8]. Cell niche can be simply changed by using various EV cell sources, different cell ratios, genetically–modified cells, priming and pre-conditioning [9–12].

**Fig. 1:**
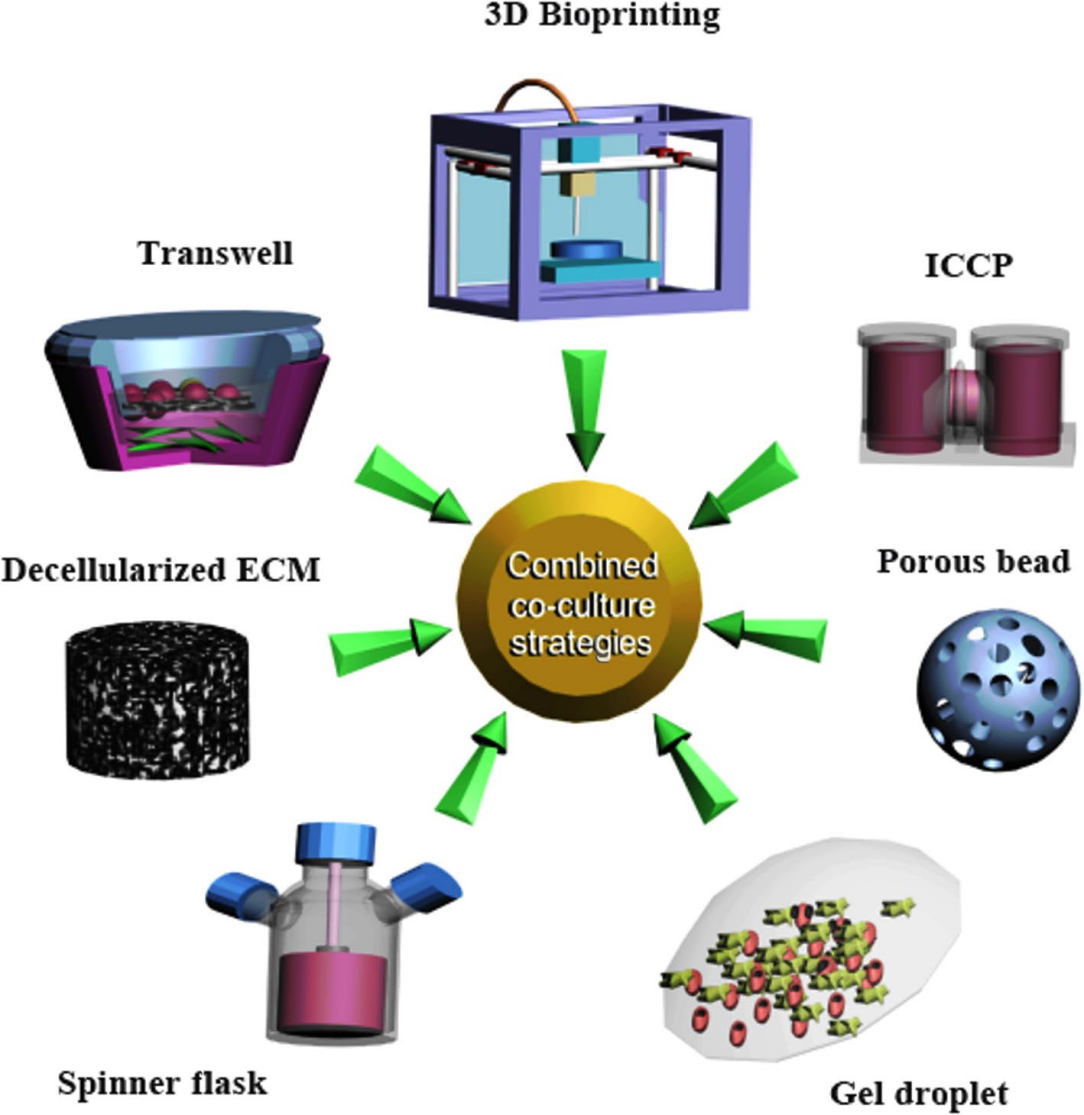
Schematic diagram that shows a number of techniques that can be used in co-culture engineering strategies (MSC: green, chondrocyte: red)

The co-culture of cells provides the membrane-to-membrane (cell–cell) interactions, cell-extracellular matrix (cell-ECM) as juxtacrine signaling, and paracrine secretion interactions such as EVs [13]. Beneficial interactions between human bone marrow mesenchymal stem cells (MSCs) and osteoarthritic chondrocytes (OACs) enhanced the MSC chondrogenesis and stimulated OACs to partially resume the lost chondrogenic phenotype [14]. Kim et al. have also shown that the direct co-culture of human synovium-derived stem cells (SMSCs) and chondrocytes enhanced the in vitro chondrogenesis [15]. Later, Han et al. observed that the co-culture of MSCs and chondrocytes with a transforming growth factor-β (TGF-β)-supplemented medium not only increased in vitro chondrogenesis but also decreased hypertrophy [16]. Control of inflammation is also pivotal to the success of OA therapeutic strategies [17]. It has demonstrated that co-culture of SMSCs enhanced cell proliferation and inhibited the inflammatory activity of osteoarthritic-like chondrocytes. Basically, compared to mono-culture systems, co-culture promotes chondrocyte growth and MSCs differentiation, decreases hypertrophy and the loss of collagen VI, improves ECM synthesis and inhibits secretion of inflammatory cytokine [7, 18]. Recent findings have proved that EVs, as an essential part of paracrine secretion, play a vital role in the reciprocal relationship during cell co-culture [6, 14, 19–21]. Diao et al. showed that the co-culture conditioned medium induced MSC chondrogenesis by upregulation of *Sox9*, *Acan* and *Col2A1* genes, increasing protein expression and Glycosaminoglycan (GAGs) production [14]. Therefore, co-culture engineering makes chances for a customized therapeutic application in OA (Fig. 1). Co-EVs is helpful for improving an OA treatment [22]; however, there are some parameters that influence its efficiency, including selecting proper cell culture methods, primitive cell numbers, type of cells, and co-culture ratios [19, 21–25] (Table 1). Here, we introduce the various co-culture systems and discuss their potential benefits and limitations for harvesting customized EVs.

**Table 1:** Co-cultured cells and their co-culture systems in cartilage research

MSCs	Cho/ OAC	Co-culture system	Cell culture type	Ref
BMSC	OAC	Mixed pellet culture	static	[26]
AMSC	OAC	Bi-layer type or the other cell culture media & Co-culture mixed in hydrogel	static	[27]
BMSC	OAC	3D co-culture with collagen microencapsulation	static	[14]
SMSC	Chondrocyte	Mixed pellet culture	Static	[15, 16]
BMSC/AMSC	AC NC	Free and perfused scaffolds	Dynamic	[19]
BMSC	Chondrocyte	Mixed co-culture on scaffolds	Static	[28]

## Direct co-culture systems

In this system, two or more distinct cell types are mixed in the 2D or 3D culture in the same environment that affect each directly (adjacent cells) and indirectly (distant cells) [6, 13]. The direct mutual effects are caused by the cell–cell contact and cell–ECM interaction (chondrocyte secreted ECM), as well as paracrine secretion; therefore, they may sense adjacent cells at short distances and create a reciprocal effect. Indirect mutual effects include only paracrine secretion in non-adjacent cells [29].

In this co-culture system, all three types of interaction exist between cells, hence it provides a high engineering potential for producing enriched engineered-EVs. Various types of direct co-culture considering their potential for harvesting engineered Co-EVs are discussed below.

### Direct 2D co-culture systems

In this type of co-culture, the physical contact of the cells is usually limited to a 2D contact, though they interact with each other through paracrine secretions. This co-culture system provides all three types of cell interactions in a 2D condition. This system includes a conventional 2D co-culture and microcarriers in the bioreactor that can produce an engineered Co-EV for the EV therapy.

#### Direct conventional 2D co-culture systems

This co-culture system is the simplest and cheapest one. In this method, petri dishes or conventional flasks are used for co-cultivation [30]. Taghiyar et al. have co-cultured mouse bone marrow MSCs (BMSCs) with rat mature chondrocytes at different ratios, including 1/1, 1/2, and 2/1 in conventional flasks. They observed that chondrocytes practically produced more chondrogenic signals that resulted in the higher MSCs cartilage differentiation by increasing the chondrocytes to MSCs ratio [30]. Mak et al. have studied the co-culturing of infrapatellar fat pad-derived passage zero (p0) adipose tissue-derived mesenchymal stem cells (AMSCs) with p0 articular chondrocytes derived from Kellgren–Lawrence Grade-III/IV osteoarthritic human knee joints in monolayer directly in T25 flasks [31]. The result showed that expression profiles of chondrogenic genes in a co-culture system were greater than in a monoculture system. Furthermore, the chondrogenic gene expression level is reduced with the enhancement of AMSC-to-chondrocyte (1:1, 10:1, and 100:1) seeding ratios. They suggested that during a co-cultivation, juxtacrine signaling, and paracrine effects pathways might be involved between ASCs and chondrocytes [31]. This system as a simplest type of co-culture engineering affects the properties of Co-EV for OA treatment by selecting different cell sources of MSCs, chondrocytes and their various ratios.

### Direct co-culture 2D/3D system by microcarriers in bioreactor

The use of microcarriers in this co-culture system allows cells to adhere onto the surface of small suspended beads in a conventional stirred-tank bioreactor, spinner flask, and shake flasks. This method is similar to the conventional 2D culture method in that the surface of traditional simple beads allows a 2D interaction for the cultured cells. At the same time, macroporous beads provide a dynamic system like a 3D culture system. In general, the surface area of these beads is much wider than the surface of conventional 2D culture. It has been shown that the EV yield of MSCs cultured on the microcarriers is greater than 2D culture systems [32]. This procedure is amenable to large-scale cell production, especially by using macroporous microcarriers in comparison with the conventional 2D cell culture system [33]. It assumes that this co-culture system has a high potential for a large-scale engineered Co-EV harvesting by different cell types.

### Direct 3D co-culture systems

The direct 3D co-culture system provides all three types of interactions. Since the 3D culture condition is closer to the natural physiological environment than the 2D culture, the 3D spatial architecture of EV cell sources affects their EV cargos [34]. Researchers have previously demonstrated that EVs derived from 3D co-cultured cells are enriched with enhanced therapeutic effects [9, 13, 19, 21]. The 3D co-culture system improves chondrocyte proliferation, MSC chondrogenic differentiation, reduces chondrocyte hypertrophy and results in functional engineered-cartilage. Against 2D systems, 3D systems can also affect EVs cargo; for example 3D systems upregulated microRNA (miR) expression and secretion [35] (Table 2). Enhancing the miRs content of EVs during a 3D culture improved their therapeutic effect on the OA treatment [35, 36]. It has also been shown that making a uniform spheroid from MSC elevated their anti-inflammatory and immunomodulatory effects [37]. A 3D culture, as an engineering approach, also increases the quantity of the EV production [38], so that it would be a more efficient method for the production of engineered Co-EV for therapeutic aims.

**Table 2:** The advantage of 3D co-culture compared to 2D monoculture in the cartilage field

Challenges of cartilage repair and OA treatment	2D mono-culture systems	3D co-culture systems	Reference(s)
Chondrocyte phenotype	Dedifferentiation	Prevention of dedifferentiation	[23]
GAG production and upregulation of chondrogenic markers	Low	High	[14]
Chondrocyte hypertrophy	High	Low	[16, 39]
Chondrocyte proliferation	Low	High	[19]
Chondrogenic differentiation of MSC	Low	High	[9]
Anti-inflammatory and immunomodulatory effect	Low	High	[37, 40]
EV yield	Low	High	[22, 32]
miRs in EVs	Low	High	[35]
Capacity for engineering and functional outcomes	Low	High	[21, 39]

#### Direct 3D co-culture systems by co-aggregation

Aggregation and coaggregation are methods of EV engineering, and harvesting engineered Co-EV [22, 38]. Cells intrinsically tend to stick together and form aggregates when they are cultivated on the specific condition, such as less hydrophilic surface or non-adhesive surfaces. This expected character is observed in different containers, including spinner flasks, stirred vessels, and multiwells. Adherent cells like MSCs, have a strong ability to bind to each other, grow, proliferate, and form aggregate. In addition, mixing and co-culturing two or more cell types also lead to their co-aggregates in such conditions [22, 41]. It is quite obvious that the dynamic 3D culture facilitates these processes. Recent study demonstrated the elevated quantity and quality of EVs produced by the co-aggregation method [22]. The study has shown that no necrotic nuclei were observed in the created aggregates with a diameter of less than 100 μm [42]. The aggregate size and diameter can be controlled by adjusting cultivation time and the cell seeding density in static cultures. In addition to cultivation time and the cell seeding density, the rotation speed must be controlled in dynamic culture systems [22, 43]. Direct 3D co-culture systems by co-aggregation have static and dynamic types that are reviewed below.

##### Direct 3D co-culture by static cluster-based self-assembly

The most common cluster-based self-assembly method for co-aggregation is hanging-drop [44]. In the simplest form of this method, drops of a cell mixture of the culture medium suspension are put on the inner surface of petri dish lids and hung. The cells spontaneously will be gathered under a gravity with the self-assembly near the tip of the drops and formed aggregates. The hanging drop technique could produce the uniform size aggregates and/or co-aggregates. However, the efficiency of this method is low, due to the use of a small amount of conditioned medium. To overcome the challenge of traditional hanging drops, a microfluidic-based hanging-drop culture system for MSCs has been developed in a tapered tube. It can augment the stability of droplets by improving the rate of liquid exchange [45]. Moreover, the microfluidic-based hanging drop as a new co-culture system could significantly increase a Co-EV harvesting rate.

Various types of ultralow adhesive surfaces cell culture plates including flat bottom, round bottom, micro pattern array, and thermal responsive surface can also be used to form aggregate or co-aggregate [44]. The round-bottom multi-well plates are widely-used because they create more uniform cell masses in terms of shape and size compared to flat-bottom plates. Due to the need for cost-effective high-scale generation of homogenous co-aggregates, diverse microwell arrays are produced from micro patterned agarose, polydimethylsiloxane, or polyethylene glycol hydrogels [46–48]. Polypropylene tubes are commonly used to prepare small amounts of micromasses [11]. Chitosan and chitosan-hyaluronan membranes or thermally responsive surface membranes can be used for co-aggregate formation. Aggregates produced by these methods are heterogeneous [49, 50]. It should be noted that these techniques have not been used in MSC/chondrocyte co-culture yet. The use of external forces, such as magnetic force has been employed in MSC aggregation for chondrogenic differentiation in a magnetic levitation model [51]. The electric field applied for mammalian T-cell aggregation [52] could also be used for co-aggregating MSCs and chondrocytes.

##### Direct 3D co-culture by dynamic collision-based assembly

In this method, a co-aggregate formation is induced by centrifugation or mixing. This mixing is mediated by shaker platforms, spinner flasks, rotating wall vessels (RWVs), and stirred tank reactors (STRs) [44]. In a study by Giovannini et al., human articular chondrocytes (hACs) and human MSCs were co-cultured for 3 and 6 weeks at various ratios, including 50–50, 25–75, and 10–90. The hACs (100%) and MSCs (100%) were considered as controls. A total of 5 × 10^5^ cells per pellet were centrifuged in 15 ml polypropylene tubes to form a micromass. They observed that chondrocytes with an innate chondrogenic potential failed to induce MSCs to undergo full chondrogenesis, with or without external chondrogenic stimulants TGF-β1 and dexamethasone after 6 weeks. They demonstrated that MSCs did not contribute to the proteoglycan deposition and hACs failed to stop the hypertrophy of MSCs induced by the chondrogenic stimuli. They found that the effect of hACs on MSCs has been only limited to early signs of neochondrogenesis [11]. Acharya et al. centrifuged 4 × 10^5^ human BMSCs or AMSCs with hACs or nasal chondrocytes (hNCs) in 1.5 ml conical polypropylene tubes to directly form co-aggregates. They also co-cultured human BMSCs and hACs in a transwell plate as an indirect co-culture method for 3 weeks. The direct co-culturing by pelleting of 25% of hNCs or hACs with 75% BMSCs or AMSCs led to an up to 1.6-fold increasing glycosaminoglycan content than other relative percentages of these cell types, but this result was not obtained in the transwell model. Hence, they suggested a direct reciprocal interaction between MSCs and chondrocytes during co-culture; MSCs induced chondrocytes proliferation, and chondrocytes improved MSC chondrogenesis [19]. Accordingly, it seems that the co-aggregation method could lead to harvesting high potential engineered EVs with a proper yield. However, the types of cell sources, age of cell donors, the number of primary cells, the co-culturing method, the duration of co-aggregate culture in the laboratory condition, and the duration of follow-up could affect the outcome.

Using a spinner flask is another convenient strategy for the relatively large‐scale production of cellular aggregates [53]. Spinner flasks provide a 3D dynamic suspension culture which induces the co-aggregation of articular chondrocytes, and MSCs [22]. It is obvious that the ratio of cells in a co-culture and the conditions of a cell mass formation affect the co-aggregate properties and the resulting EV. For example, He et al. prepared an aggregation of bovine articular chondrocytes (bACs) and rabbit bone marrow‐derived mesenchymal stem cells (rMSCs) and their co-aggregate (1:1 rMSCs/ bACs, 40 rpm) by suspension culture in a spinner flask. Both rMSCs and bACs displayed an increased aggregation rate and aggregate size by reducing agitation rate (60, 50, and 40 rpm) and enhanced cell inoculation density. Furthermore, an extended culture time led to the formation of aggregates with larger size. According to the gene analysis, the expression of both genes, integrin β1 and cadherin, may be involved in the aggregation process that codes cell-ECM binding and cell–cell contact proteins, respectively [53]. More recently, our research group formed a co-aggregation of rabbit BMSCs and ACs (3:1 MSCs/ACs, 40 and 45 rpm) by spinner flask and used harvested EVs in the treatment of OA in a rat model. Our results showed that the co-aggregation method elevates the Co-EV yield and its therapeutic effect compared to EV isolated from MSC or chondrocyte aggregates [22].

A dynamic 3D culture tends to fall into ranges of diameter of aggregates/co-aggregate that these ranges are influenced by controlling the number of primitive cells and their ratio, and the bioreactor rotation speed (Table 3). Since the most important goals of current research is to increase the secretion of Co-EV by co-culturing, it seems that a too large aggregation is hosted of internal necrotic cells [44] that result in a low EV yield.

**Table 3:** Different ratios and methods of cell co-aggregating

Dynamic collision-based assembly procedure	Co-culture cells	Co-culture cell ratio (MSC/Cho)	Revolutions Per Minute (rpm)	Primitive cell density cells/mL	External chondrogenic stimulants	Direct/indirect co-culture	Ref
Centrifugation	hAC/ hMSC	1:1, 3:1	-	5 × 10^5^	TGF-β1 & dexamethasone	Direct	[11]
Centrifugation	BMSC or AMSC/AC, NC	3:1	-	4 × 10^5^	-	Direct & Indirect	[19]
Spinner flask	bAC/rBMSC	1:1	40	2 × 10^5^	-	Direct	[53]
Spinner flask	rBMSC/rAC	3:1	40 & 45	5 × 10^5^	-	Direct	[22]

The shear stress is one of the most significant factors in dynamic culture systems. Generally, the average shear stress has a reversed relationship with the average aggregate size but a direct relationship with fluid rotation [43]. Dynamic culture induces different levels of shear stresses that affect cellular behaviors [54] such as cell proliferation and morphology [55]. Although high rates of shear stress damage cells, it is demonstrated that a mid-level shear stress is necessary for cell growth because it facilitates extracellular protein secretions and improves cell diffusivity during the culture [56]. In a dynamic culture system, the minimum and optimized speed for the particles to flow through the mixture is 30 and 60 rpm, respectively [57]. The shear stress at 60 rpm is approximately 4 dyne/cm^2^ [58]. Mechanical forces induced by shear stress are important in self-renewal signaling pathways regulation in cancer cells that result in upregulation of shear stress-related gene including Egr1, Ap1, Epcam, Klf8, and Klf2 [58]. In addition, shear stress regulates endothelial membrane-shed microparticles release [59]. Jo et al. have developed a microfluidic device to exert shear stress and improve the yield of EVs [60]. Furthermore, Yan et al. confirmed that mechanical stimulation of UC MSCs increased the exosome yield and its biological effect for cartilage repair, which could be related to the high expression level of the LncRNA H19 in exosomes [61]. Also, Chung et al. have observed that shear stress controls the landscape of miRs in endothelial Cell-derived small EVs [62]. Shear stress in the optimal range (around 60 rpm) can increase the yield of EVs and improve their therapeutic effects in cartilage repair. However, comparative research is needed to achieve the particularly appropriate rotation speed for optimally sized co-aggregate formation and secreted Co-EV and their cargo.

#### Direct 3D co-culture of different cell types aggregates

Direct co-cultivation of separately formed aggregate from two or more different cell types is another strategy for harvesting engineered Co-EVs. These direct co-cultured cell aggregates could prepare a cell–cell contact, only via cell membrane contacts and paracrine interactions. Their harvested EVs' properties most probably is different from the Co-EVs harvested from the mixed co-aggregate of these cells. Understanding the differences between these two approaches would pave the way for co-culture engineering in order to customize EV engineering.

#### Direct 3D co-culture systems by tissue engineering

Tissue engineering uses a combination of cells, scaffolds, and signals to create desired cell niches, so that it can provide tremendous potential for customized EV harvesting for functional tissue regeneration. In this approach, two or more cell types are seeded in a common or different scaffold; these cells interact with the scaffold as a special ECM. Different types of scaffolds such as biochemical and physical factors affect the cell behavior and their interactions. In this method, different types of cells interact with all three types of interactions. Due to the wide variety of scaffold types, cells and signals, there is a wide engineering co-culture possibility for the engineered EV production, which could influence the quantity and quality of a Co-EV production.

The tissue engineering through stem cell-based co-culture has been used for the treatment of bone, heart, liver, nerve, kidney, lung [13], and cartilage [63]. Studies have proved that tissue engineering enhances a chondrogenic phenotype in monoculture and co-culture of articular chondrocytes and MSCs [64]. Since tissue engineering reproduces a quasi-natural niche of cartilage tissue via the co-culture engineering, it could provide engineered EVs for the OA therapy. This approach can be scaled up for the efficient Co-EVs production. The genetically-modified cell sources can also be employed in tissue-engineered constructs for co-culture engineering to produce therapeutic customized EVs and treat articular cartilage disorders. Zhang et al. mixed MSCs and transgenic chondrocytes with the alginate hydrogel for cartilage tissue engineering in a 3D environment. They have delivered a transforming growth factor-β3 (TGF-β3) gene to the chondrocytes with adenoviral vectors. They observed that the release of TGF-ß3 from transgenic chondrocytes induced the chondrogenesis of SMSC, and also the chondrocyte phenotype was preserved from a presumed dedifferentiation process [23]. Another research group co-transduced SMSC by co-delivery of lentiviral vector containing TGFβ3 gene and adenoviral vectors of the gene for small hairpin RNA which facilitated SMSC chondrogenesis and suppressed Col I expression in SMSC and minimize fibrocartilage formation, respectively [65]. They concluded that these co-transduced cells generally displayed an optimal efficacy. These properties of genetically-engineered cell sources could be reflected in their secreted EVs. In addition, the overexpression of tetraspanins [66, 67] and Rab GTPase [42] in MSCs and chondrocytes could be effective in high scale engineered Co-EV production for a cartilage repair (Table 4).

**Table 4:** Different scales for cell co-cultivation

Scale	Procedure	Application	Ref
Small	Ultralow adhesive multiwell plates	Research	[44]
Medium	Shake flasks, spinners, roller bottles, wave bags, or bioreactor systems including microcarriers and hollow-fiber bioreactors	Research and Clinic	[34]
Large	Stainless steel bioreactors (up to 20,000 l scale), platform-rocker wave bags (up to 500 l scale), or even disposable bioreactors (up to 2,000 l scale)	Clinic	[4]

## Indirect co-culture system

**Fig. 2:**
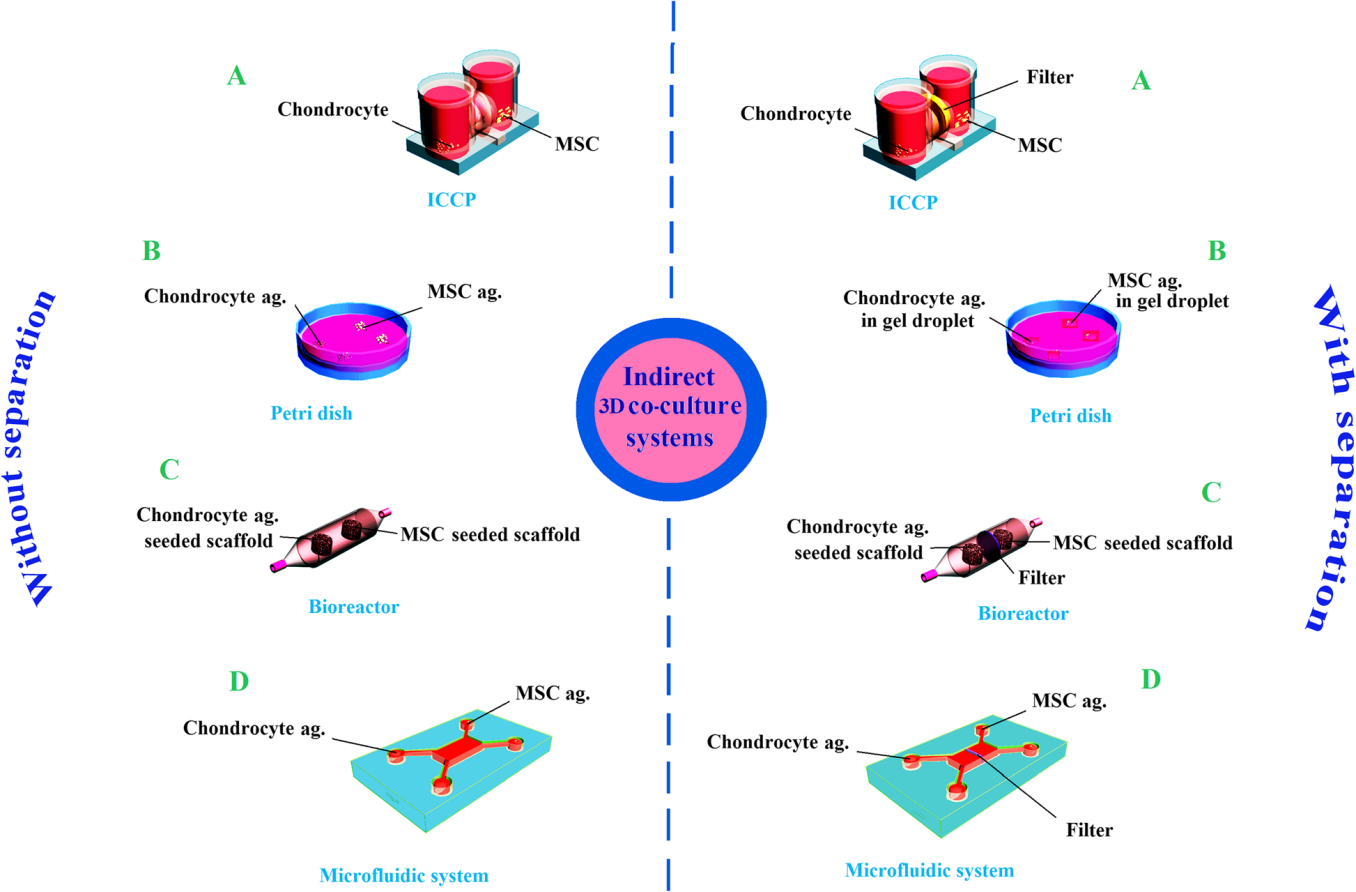
Schematic diagram represents some techniques of indirect 3D co-culture system engineering (ag: aggregate). **A** 2D co-culture of MSC and chondrocytes in ICCP. **B** Co-culture of MSC and chondrocyte aggregates in gel droplets in the petri dish. **C** Co-culture of MSC and chondrocyte seeded onto scaffolds in the bioreactor. **D** Co-culture of MSC and chondrocyte aggregates in the microfluidic system

Indirect co-culture systems contain two or more distinct types of cells that are co-cultured in a 2D or 3D culture condition. In this system, the environment could be either unseparated or divided by a physical separation via filter (in a transwell, horizontal co-culture plate, microfluidic systems, and bioreactor systems) or gels as a solid separator (cells in gel droplets in a petri dish or microfluidic system) [6] (Fig. 2). These different technical conditions apparently lead to a different therapeutic outcome of engineered Co-EV cargos.

In the indirect co-culture systems with or without separation, the interaction between co-cultured cells does not occur via a cell–cell interaction and their interaction is restricted to their paracrine interaction [29].

Paracrine interaction can be controlled by environment separators. The separator’s features determine the molecules and particles that exchange between the separated parts of the environment. In other words, co-cultured cell interactions are merely limited to EVs and selected soluble factors. For example, by the selection of proper filter size, the interaction could be limited only to a certain size range of soluble factors which have similar biochemical properties. Gregg et al. have used a tissue culture system for indirect co-culture. They have placed synoviocytes on the bottom of the transwell and have suspended equine cartilage explants in a transwell insert by a low–protein binding polyester membrane with a pore size of 3 μm. The membrane also has gaps in its peripherals to let the explants share a common medium with synoviocytes. Their results showed that synoviocytes secreted mediators could protect matrix GAG metabolism from the degradative effects of IL-1beta. [68].

Indirect co-culture system enables us to study the effect of the paracrine interaction without a cell–cell physical contact or even study the effect of soluble factors without an EV interaction. In addition, we could explore how the separators affect the quality and quantity of secreted Co-EVs in comparison with a Co-EV production in the separation free condition. Hence, this method is helpful in the most precise evaluation of the mutual molecular interaction mechanism of co-cultured cells through their paracrine secretions.

Overall, since the types of interaction between cell sources impact their Co-EV cargo, there is possibility of harvesting various EVs from different engineered co-culture with controllable paracrine interaction in a variety of conditions and cell sources. In the following, we describe different types of indirect co-culture systems.

### Indirect conventional 2D co-culture systems

This system is the simplest approach for the indirect co-culture of two or more cell types (Fig. 2, A). In this case, the cultured cells do not show a cell–cell contact in response to the juxtacrine signaling, but interact through paracrine secretions via the culture medium, with or without separation. Similar systems to indirect 2D co-culture systems were used as MSCs and chondrocytes were cultured in distinct gels but in the same wells. Results have revealed improvement in neocartilage formation and decreased hypertrophy compared to MSC-alone hydrogels [39]. It is expected that the separation or non-separation of co-cultured cells may affect the therapeutic potential of harvested Co-EVs.

A novel cell-culture chamber has been developed with a transverse connection for a co-culture container usage, which refers to the “interactive co-culture plate (ICCP)” that provides clear observations of cell morphology and cell behavior with a microscope [6]. The ICCP is useful for the indirect cell interaction engineering and studying their behavior. It is apparent that this system has a high potential to improve the quantity and quality of harvested engineered Co-EVs.

On the other hand, an ICCP transverse connection can be separated by a filter and changed into a 2D co-culture with a separation [6]. This system has a high potential to control co-cultured cell interaction because physical, chemical and biological properties of the filter determine its permeability to various paracrine secretions such as EVs. The separator can be applied as an engineering tool for a co-cultured cell interaction, and in turn, would result in the production of Co-EVs by engineered cell interaction.

### Indirect 3D co-culture systems

This type of co-culture is useful for studying the interaction of two or more separate cell aggregates. This system can be applicable to reconstituting the tissue-like structures (similar to in vivo) that are distant from each other. In addition, it could be useful for obtaining customized engineered Co-EVs that harvest from a separate aggregates interaction. In this indirect co-culture system, there is a 3D cell–cell interaction in a cell micromass or cell-seeded construct, yet in a different microenvironment. Accordingly, there is no possibility of a physical contact or juxtacrine signaling, and their interaction is only limited to paracrine secretions (Fig. 2, B). Due to the use of the 3D culture method in a co-cultivation system, it is believed that the quality and quantity of an engineered Co-EVs production change significantly [69, 70]. It is obvious that engineering the type of a cell interaction through a co-culture engineering lead to the production of various engineered EVs from human MSCs with different therapeutic outcomes [38]. Of note, aggregation and tissue engineering approaches are two EV engineering procedures for the harvesting engineered Co-EV for the OA treatment.

#### Indirect 3D co-culture systems by aggregation

In this system, at the first step the desired cells are aggregated, using one of the cell aggregate methods, and then used for indirect co-cultivation. Here, the separate aggregations of different cell types interact only through paracrine secretions (Fig. 2, B, C and D). This procedure includes non-separated or separated types of indirect co-culturing aggregations. Rickert et al. indirectly co-cultured micromass pellets and periosteal explants. Periosteal explants were placed at the bottom of the transwell and micromass pellets above. They used a 1 µm porous membrane for separation. Their results demonstrated that periosteum/chondrocytes co-culture altered the expression profile of matrix metalloproteinases [71].

We suggest that in an indirect 3D co-culture, two or more separate aggregations from different cell types can be co-cultured together. This interaction type could produce special cargoes in an engineered Co-EV. For example, this system might be applicable as a microfluidic system, to study the therapeutic properties of harvested EVs from a culture medium of separate aggregates.

In the second type of the indirect 3D co-culture, aggregation of different cell types are isolated from each other by a physical separator like a filter in the same environment (Fig. 2). The physical and chemical properties of a filter could control and limit its exchange and interaction (Fig. 2, D). By selecting the type of filter, the interaction between the aggregates could be engineered during co-culture and produced specially engineered EVs. For instance, a filter with a small pore size could limit molecular interaction between aggregates only to small EVs and soluble factors. Therefore, it can be expected that different filters create different interactions, and consequently different EVs and therapeutic properties.

#### Indirect 3D co-culture systems by tissue engineering

The variety of scaffolds, signals, and cells provides a wide engineering design possibility to produce Co-EVs, which influences the quantity and quality of these engineered Co-EVs. There is also a possibility to harvest Co-EVs from condition media following the interaction of different cells with scaffolds and use them to improve cartilage tissue engineering.

**Fig. 3:**
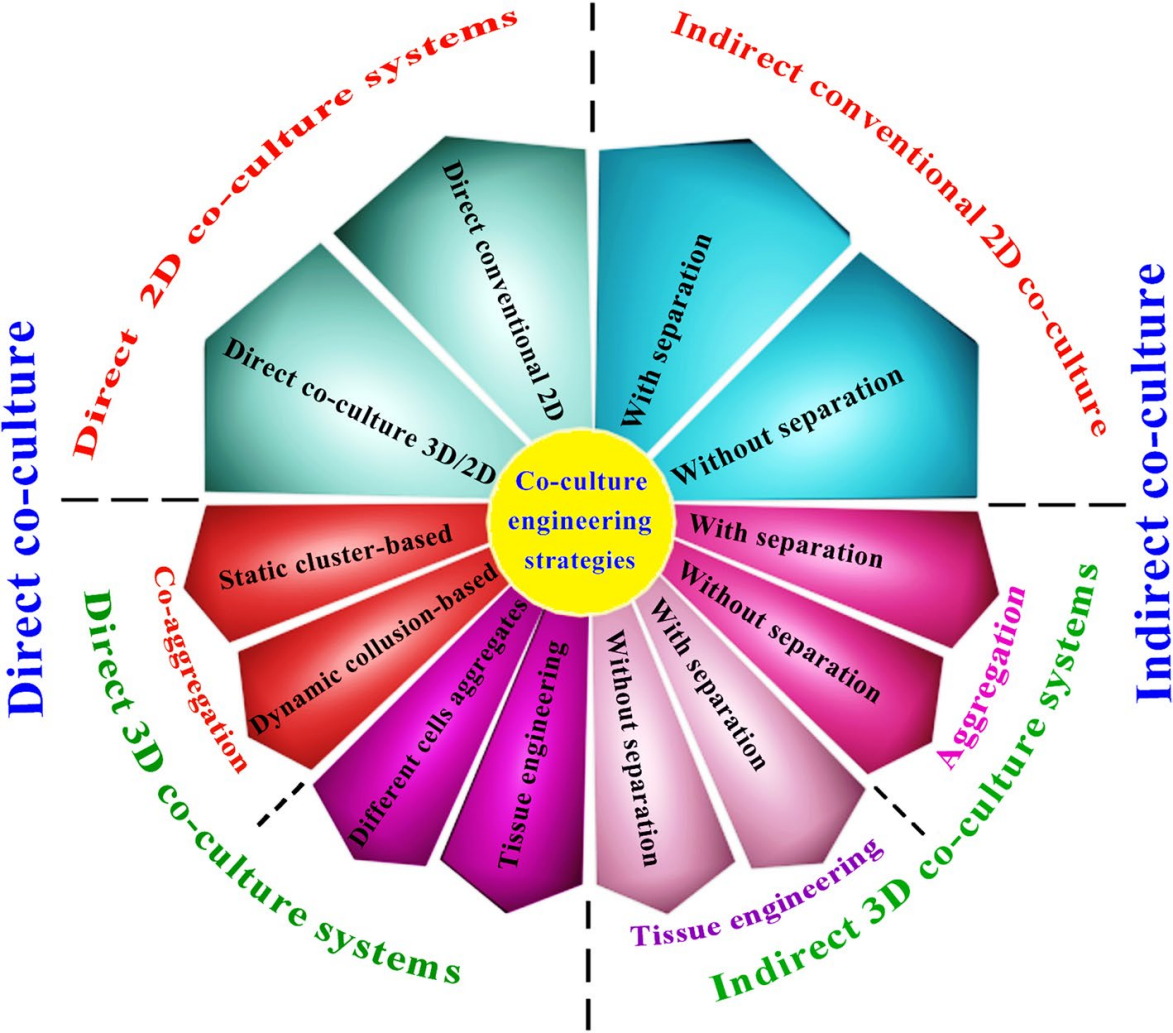
Schematic diagram that shows combined co-cultured strategies including various direct, indirect, 2D, and 3D systems

In the first type of the 3D co-culture system, there is no separator and the scaffolds are seeded by different cell types and placed far from each other; therefore, they interact easily through the culture medium (Fig. 3). Levorson et al. have studied the impact of indirect contact in co-cultures of MSCs/chondrocytes. They aimed to improve the cartilage-like ECM deposition within nonwoven fibrous poly (e-caprolactone) (PCL) scaffolds. Their results showed promotion of ECM synthesis by chondrocytes that in turn induced MSC chondrogenesis [63]. An alternative plan in such studies can be collection of conditioned media, isolation of Co-EVs and evaluation of their therapeutic effects.

In the second type of the indirect 3D co-culture systems, separators are placed among the cell-seeded scaffolds to control paracrine exchanges (Fig. 3, C). A range of separation tools, including different filters or gel droplets, can be employed. Xu et al. have designed a 3D dynamic co-culture system and co-cultured rabbit chondrocytes-laden alginate beads and rabbit MSCs-laden alginate beads (1.8 w/v%) in a spinner flask. They observed this co-culture system improved chondrogenesis more than monoculture MSCs [72]. Similarly, Kim et al. have studied MSC/chondrocyte-laden hydrogel constructs with a ratio of 4:1. They used the juvenile bovine chondrocyte and adult bovine MSC in a different zone of the hydrogel. Their results showed the interaction between the chondrocyte and MSC enhanced viability, chondrogenesis, and ECM production. They claimed that the chondrocyte and MSC proximity is a requirement for these outcomes. Moreover, they indicated that an EV transfer blockage stops the synergistic effect of a co-culture. This study identified EVs as the main tool of interactions in this co-culture strategy [21]. Diao et al. co-cultured MSCs and OACs, which had already been encapsulated in collagen. They have prepared entrapped microspheres of hMSCs and hOACs. Their results showed that the hOAC-conditioned medium and a co-culture with microspheres of hOACs are both chondroinductive to hMSCs microspheres. The co-culture of hOACs microspheres with hMSCs microspheres with or without chondrogenic pre-differentiation partly renewed the chondrogenic phenotype of hOACs. They reported reciprocal interactions between hMSCs and hOACs during a co-culture procedure [14]. In addition to scaffolds and different cell sources, various signals, such as growth factors, can be applied in this system, and its effects on the quantity and therapeutic properties of EVs obtained from co-cultivation can be investigated.

Since secreted EVs could pass from even condensed matrices, the conditioned medium containing the engineered Co-EVs [73] can be isolated. Extracted EVs might have different therapeutic effects that require further studies to prove. Albeit, given that cells are gel encapsulated, these systems should be evaluated in terms of the EV production efficiency compared to other systems.

## Combined co-culture systems

Each co-culture system could meet some needs of research or clinical applications; therefore, a combined co-culture strategy is needed to obtain more effectively enriched Co-EVs for EV therapy. In this regard, several studies have applied scaffolds in bioreactor systems for the co-culture of MSC/chondrocyte in order to improve chondrogenesis. For example, Levorson et al. examined the impact of indirect and direct cell–cell contacts in co-cultures of MSCs/chondrocytes. They aimed to improve the deposition of the cartilage-like ECM on nonwoven fibrous poly(e-caprolactone) (PCL) scaffolds. In static cultures, the indirect co-cultured group had significantly greater quantities of the glycosaminoglycan and collagen than the direct co-cultured group. They observed that indirect co-culture groups improved the ECM contents. They suggested that chondrocytes induce the chondrogenesis of MSCs via a high ECM synthesis within bioreactor culture. This study attributed their results to secretory factors from the cells [63].

It is possible to evaluate therapeutic effects of EVs harvested from co-culture of different cells that are separately cultured in 2D and 3D systems. For example, Yang et al. co-cultured the chondrocyte pellet with monolayer culture of MSC in a transwell with a 0.4 μm pore size membrane. They demonstrated that an indirect co-culture of BMSCs and ACs reduced hypertrophic markers and led to a decrease in the endochondral bone formation induced by exogenous stimulators [74]. It is expected that the EVs harvested from the conditioned medium of this co-culture have special therapeutic properties that need to be explored. Leyh et al. have designed a “tri-culture” model, where BMCSs/ACs have been cultured with a 1:1 ratio embedded in fibrin gels, and then the construct placed onto the surface of cartilage explants in a chondrogenic medium. Their results have proved that soluble factors like pro-inflammatory cytokines were released from OA cartilage explants, and partially prevented the collagen synthesis, and inhibited the differentiation of BMSCs to the chondrocytes [75]. In addition to soluble factors, the therapeutic effects of EVs released from OA cartilage explants and also EVs harvested from their condition medium are suggested to be assessed on the OA treatment. Customized priming of MSCs and chondrocytes through their co-culture with specific cells and tissue explants might impact the content of the extracted EVs and their therapeutic properties. In another experiment, Dahlin et al. investigated the chondrogenic potential of co-cultured bovine articular cartilage cells and rabbit bone marrow MSCs in biodegradable electrospun PCL scaffolds at low TGF-β3 concentrations. Their findings proved that co-cultures of ACs and MSCs need a low level of TGF-β3 to obtain an equivalent level of the in vitro chondrogenic differentiation in comparison with each monoculture condition, via augmentation of TGF-β3 effects [28].

According to the results of mentioned studies, it seems that a co-culture engineering with a combined strategy could provide a higher level of EV engineering, by using different potentials of co-culture techniques, methods, and various cells (Fig. 3). It is expected that this strategy leads to the production of enriched and customized Co-EVs for therapeutic applications.

Various co-culture systems discussed in this review may lead to Co-EVs production with different quantities and qualities. For clear judgment, we need to compare them under the same conditions in terms EV yield and therapeutic effects. But overally, harvesting EVs through 3D dynamic co-culture and tissue engineering needs higher cost, technological knowledge, and modern tools than 2D and static culture methods, providing a unique possibility for advancement in EV therapy. Finally, it should be mentioned that each of these systems can answer a specific research question and would pave the way for the future pre-clinical and clinical research.

## Limitations in vitro cartilage research & co-culture engineering

Although chondrocyte and MSC cultivation have provided a very valuable and unprecedented contribution to biological and medical cartilage research for optimal and more effective use, their limitations should be considered in research and clinic. Solving the problems and limitations of chondrocyte culture can lead to the correction of their phenotype and function and produce enriched EVs to use for preclinical and clinical research.

One of the most important limitations is that chondrocytes lose their phenotype and undergo dedifferentiation upon an in vitro culture [76]. Although the co-culture of chondrocytes and MSCs prevent the loss of the chondrocyte phenotype [14], it is noteworthy that during a dynamic co-culture, the chondrogenic phenotype and interaction of ACs with other co-cultured cell types can be modulated by flow rate of medium and shear stress [9].

Unlike an in vitro culture environment, articular cartilage is an avascular and hypoxic microenvironment. It seems that this issue could be considered as the incompatibility of in vitro conditions with natural conditions that affect clinical outcomes. Therefore, reconstruction of the hypoxic state of articular cartilage during in vitro experiences [77, 78], especially in the co-culture, would be very useful. According to recent findings, the hypoxic condition influences the quantity and therapeutic properties of engineered EVs [79–82]. For example, hypoxia-inducible factor 1 alpha (HIF1α) is upregulated during pellet co-culturing by an orbital shaker [83]. It has been proved that activation of HIF1α improves small EV secretion in human embryonic kidney cells [80]. Zhang et al. confirmed that EVs derived from hypoxia-preconditioned MSC enhanced chondrocyte proliferation and migration and reduced chondrocyte apoptosis compared to normoxia-preconditioned MSC-derived EVs via in vitro experiments and in vivo OA models [84]. They observed that hypoxia altered the miR expression level in MSC-EVs and four OA-related differentially expressed miR downregulated; hsa-miR-181c-5p, hsa-miR-18a-3p, hsa-miR-376a-5p, and hsa-miR-337-5p. These miRs stimulate chondrocyte proliferation, migration, apoptosis suppression, and eventually improve OA. Similarly, several studies have found that hypoxic conditions increase the expression level of some cartilage-related miRs, such as miR-210 [85], miR-21 [86], and miR-23 in secreted EVs [87]. miR-210 is present at both downstream and upstream of HIF‐1α [88]. Upregulation of miR‐210 in EVs derived from MSCs cultured in hypoxia [89] stimulated chondrocyte proliferation [90]. miR‐21 controls the development of OA by targeting GDF-5 in chondrocytes [91]. A feedback regulatory loop has already been revealed between HIF-1α and miR-21 in response to hypoxia [92]. miR‐23a has a critical role in cartilage homeostasis [93], and indirectly regulates cellular levels of HIF‐1α [87]. Altogether, it seems that miRs and HIF‐1α have a reciprocal regulation in hypoxic conditions. We assume that the co-culturing of MSC and chondrocyte changes miRs and HIF‐1α expression levels and in turn improve EV yield and their therapeutic effects for OA treatment.

During a chondrogenic differentiation process, MSCs tend to a hypertrophic phenotype, which prevents the hyaline articular cartilage production. Therefore, besides monoculture MSC associated with specific factors, co-culture of MSC and chondrocytes has been suggested and applied as 3D, 2D, direct, and indirect methods [64] that need more study. A recent study showed that a 3D dynamic culture system with TGF-β3 enhanced the production of potent MSC-derived EVs, which have high therapeutic potential, including migration of dermal fibroblasts and wound closure, as evidenced by comprehensive proteomic analysis [94]. It could be expected that this experiment with co-culturing could elevate their harvested EV therapeutic effects.

Dynamic 3D culture systems in comparison with static culture systems can improve nutrient supply and other relevant physio-chemical factors. In addition, dynamic 3D culture improves cell–cell and cell–ECM interactions, proliferating, and differentiation, by higher cell–cell adhesion and cell-ECM remodeling [95]. Kang et al. have shown that the bioreactor EV yield is nearly seven times more EVs per cell in 24 h compared to static conditions by designing a flat-plate bioreactor [96].

Although Co-EVs can help solve the challenges and limitations of cartilage research, EV delivery to the recipient cells is a significant challenge in their therapeutic application. Various methods are used for this purpose. In addition to the use of a hydrogel for sustained release of EVs [97, 98], 3D models were produced by microfabrication techniques containing electrospinning. 3D printing has prepared excellent opportunities as delivery systems for EVs [99]. Hence, the development of this approach could have a high potential for EV delivery by tissue engineering as an engineered EV-delivering procedure for EV therapy.

## Current status of Co-cultured EVs in clinical trials

A promising therapeutic effect of EVs has resulted in increasing the number of clinical trials related to EVs which have been well-reviewed and analyzed elsewhere [100, 101]. Thus far, around 80 clinical trial records are available on clinicaltrials.gov. However, no clinical trial has been registered regarding the administration of Co-EVs and even EVs for cartilage regeneration yet.

Since the major challenge is the cost-effective scale-up production of EVs with acceptable therapeutic properties [100], co-culture engineering, particularly 3D dynamic co-culture, is believed to overcome these challenges. The 3D dynamic culture method supports large-scale production, and co-culture engineering not only increases EV yield but also enrich the EV cargo and elevate their therapeutic effects [22, 69, 72].

MSCs-derived EVs are one of the most types of EVs used in clinical trial settings. MSCs-derived EVs are interesting due to their various properties, including enhancing proliferation, attenuating apoptosis, and modulating immune reactivity [102]. Accordingly, the 3D dynamic co-culture of MSC with other cells, such as chondrocytes, would be an appealing strategy to produce adequate EVs and improve the outcome of future clinical trials.

## Conclusion and future perspectives

In recent years, therapeutic effects of EVs have been demonstrated in various diseases, such as OA. The optimized clinical application of EVs requires elevating the quantity and quality of the EVs production. Recent studies have demonstrated the co-culture of MSC and chondrocytes and other cells have mutual benefits such as elevating the chondrogenesis, controlling inflammation, and paracrine interactions, and preventing hypertrophy and apoptosis by enrichening of their EV cargo such as miRs. It is believed that the EV engineering in combination with a co-culture engineering would be useful to elevate Co-EV properties. Co-culture engineering includes cell–cell interaction engineering, cell-ECM engineering, and paracrine interaction engineering. Therefore, co-culture engineering of different cells involved in a cartilage repair is helpful in the preparation of customized engineered Co- EVs to improve the OA therapeutic approaches.

Since the co-culture strategy has direct, indirect, static, and dynamic systems in 3D and 2D culture techniques and each of them has various sub-categories, therefore it is possible to use these various methods along with the selection of suitable co-culture cells and their priming or genetic manipulation for OA research and treatment. In addition, engineering the cultivation conditions by using different physical, biochemical, and biological factors helps us to design special niches, and could lead to the production of more effective customized Co-EVs for therapeutic applications.

To sum up, each of co-culture systems and their engineering are effective in the production of specially engineered Co-EVs for research and therapy. A comprehensive combined strategy is needed for more effective and promising treatment. In this regard co-culture engineering methods could use increasing advances in the field of 3D cell culture, high-scale cell culture, cell and tissue engineering, cell niche engineering, and EV engineering to produce customizable engineered Co-EVs for diseases treatment such as OA.

## Data Availability

Not applicable.

## Abbreviations

AMSC: Adipose tissue-derived mesenchymal stem cells
BََMSC: Bone marrow MSC
bAC: Bovine articular chondrocyte
Co-EV: Co-culture harvested EV
ECM: Extracellular matrix
EVs: Extracellular vesicles
GAGs: Glycosaminoglycan
hAC: Human articular chondrocyte
HIF1α: Hypoxia-inducible factor 1 alpha
ICCP: Interactive co-culture plate
MSCs: Mesenchymal stem cell
miR: MicroRNA
NC: Nasal cartilage
OAC: Osteoarthritic chondrocyte
OA: Osteoarthritis
PCL: Poly(e-caprolactone)
rMSC: Rabbit bone marrow‐derived MSC
SMSC: Synovium-derived stem cell
TGF- β: Transforming growth factor β
UCMSCs: Umbilical cord MSCs
